# Design and Performance Evaluation of a Wearable Sensing System for Lower-Limb Exoskeleton

**DOI:** 10.1155/2018/8610458

**Published:** 2018-09-18

**Authors:** Chunfeng Yue, Xichuan Lin, Ximing Zhang, Jing Qiu, Hong Cheng

**Affiliations:** ^1^The School of Automation Engineering, University of Electronic Science and Technology of China, China; ^2^Center for Robotics, University of Electronic Science and Technology of China, China

## Abstract

Because the target users of the assistive-type lower extremity exoskeletons (ASLEEs) are those who suffer from lower limb disabilities, customized gait is adopted for the control of ASLEEs. However, the customized gait is unable to provide stable motion for variable terrain, for example, flat, uphill, downhill, and soft ground. The purpose of this paper is to realize gait detection and environment feature recognition for AIDER by developing a novel wearable sensing system. The wearable sensing system employs 7 force sensors as a sensing matrix to achieve high accuracy of ground reaction force detection. There is one more IMU sensor that is integrated into the structure to detect the angular velocity. By fusing force and angular velocity data, four typical terrain features can be recognized successfully, and the recognition rate can reach up to 93%.

## 1. Introduction

Lower limb exoskeleton (LLE) robotic technology has been developed rapidly for the past 20 years. Three implementation fields, that is, augmentation, rehabilitation, and living support are explored. First, the human augmentation type LLE (AULLE) was developed for the military and aims at improving a soldier's weight loading ability. The fields of application extend to disaster relief and industrial transport assistance. Kazerooni et al. developed the typical first generation AULLE which is named BLEEX [1, 2]. Then, the third generation AULLE which is named HULC was developed for carrying a load of about 90 kg. Second, the rehabilitation type LLE (RELLE) was developed for patients whose lower limbs are inconvenienced [3–5]. Typical patients include those with foot drop, spinal cord injuries, and strokes. The most famous RELLE is Lokomat, which is developed by the Hocoma Company [6]. The assistive-type LLE (ASLLE) is used to assist patients with lower limb disabilities but whose upper limbs are normal. The ASLLEs assist patients to return to their normal life. They do not only assist in walking motion on flat ground, climbing on stairs, or sitting down and standing, but they also rebuild their confidence in daily life. There are three typical commercial products that have been developed: ReWalk 6.0 [7], Ekso GT [8], and HAL-5 [9]. They each have a weight of about 20 kg. Besides these, lightweight ASLLEs have also been developed to assist spinal cord injury patients, such as Phoenix 3.0 [10] and INDEGO [11]. The researchers are also trying to prove their benefit to patients who have used the ASLLEs [12, 13].

**Figure 1:**
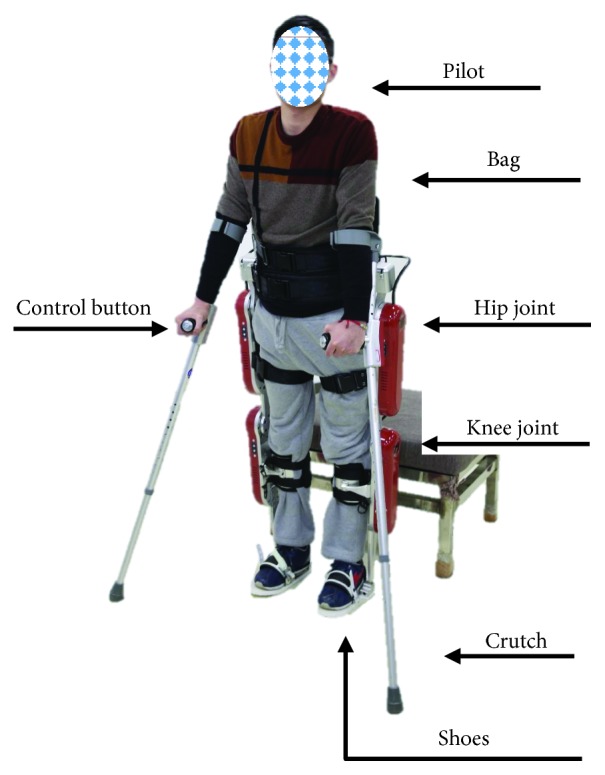
The prototype of AIDER with a SCI patient.

In our previous research, we focused on the human augmentation exoskeleton [14–16] and an ASLLE named the AIDER [17, 18] for individuals. An illustration of a prototype of AIDER with a SCI patient is shown in Figure 1. Based on AIDER and two crutches, the patient can form a stable area to keep balance. Four DC motors are installed on the hip and knee joints respectively. The patient controls the AIDER by two controllers which are installed on the grab handles of the crutches. A battery and control circuit placed in the backpack are the core part for controlling the DC motors. This paper is an extension for my previous work [19].

Avoiding harm to the patient when piloting an exoskeleton is a critical issue.  Table 1 shows the influence of three kinds of LLEs on the interaction between human, machine, and the environment. For an AULLE, the mechanical parts follow the pilot's motion and support the weight of the load. The pilot takes the initiative in the human-machine-environment system. For a RELLE, the machine controls the human motion because of the lower-limb paralysis of SCI patients. The working environment of a RELLE is very safe because the pilot is always protected by fixed support. For an ASLLE, although the potential pilots are the same as those of a RELLE, the working environment for an ASLLE is complex daily life. The safety for the pilot of an ASLLE relies on the exoskeleton's stability. Besides, patients with complete injury have lost their sensory ability. They cannot keep their balance when wearing the exoskeleton. Therefore, the ASLLE should be able to sense the human-machine state and adjust the gait trajectory for the pilot to avoid a dangerous situation.

Based on the analysis for the safety of a human-machine system, a sensing system is necessary to improve the stability of ASLLEs. The shoes are suitable components for installing sensors. In related works, researchers have used foot-sensing systems to detect gait information. Footscan is a typical commercial product which was developed by the RSscan Company. Based on this product, the relationship between the gait characteristics and foot pressure for overweight children was analyzed [20]. Besides, researchers also designed a number of wearable sensing systems to meet their research requirements. In [21], the researchers designed sensing shoes to estimate the CoM displacement continuously using an ambulatory measurement system which contains 2 IMUs and 2 6DOF force/moment sensors. Although the precision of the sensor is really high, the large size influences gait analysis resulting from normal walking. Even high precision data can be collected easily. The large size of the IMU and pressure sensor gives the pilot poor wearing experience. Liu et al. have worked on gait analysis for serval years. They developed smart shoes for human gait analysis [22, 23]. The novel point of the smart shoes is that they contain three 6-axis force sensors with 1 cm thickness. The sensors are mounted on the heel, arch, and forefoot respectively to subdivide the phase of human walking and get high accuracy gait data. Bamberg et al. developed a multiple sensing system and integrated it with shoes. The sensing system contains 6 kinds of sensors including an accelerometer, gyroscope, force sensitive resistor, bend sensor, polyvinylidene fluoride strip, and electric field sensor [24]. The multiple sensors provide redundancy gait data to ensure stability. Due to the complex environment as shown in Table 1, we intend to develop a wearable sensing system for AIDER which can not only detect human body motion but also detect the state of the environment, such as the features of the ground.

## 2. Motivation

### 2.1. User and Application Environment for AIDER

AIDER is intended for users with SCI with injury levels from T9 to T12 caused by traumatic injuries (e.g., vehicular crashing or falling from buildings) or disease (e.g., myelitis) [25, 26]. The AIDER is aimed at extending the range of activities to advance their rehabilitation programs for SCIs. Besides, walking upright makes the patients feel more confident because they can make a conversation with friends at eye level and walk like normal persons.

**Figure 2:**
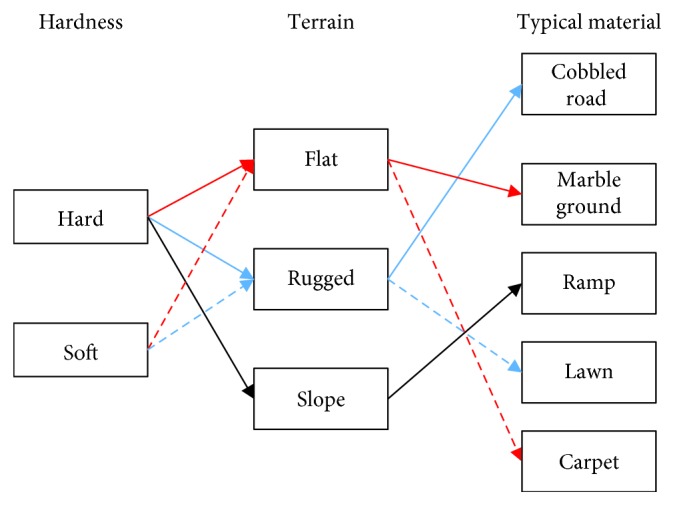
The common application environment for users of AIDER. The solid line and dotted line stand for hard and soft features, respectively; red, blue, and black denote flat, rugged, and slope features, respectively.

As illustrated in the Introduction, the target environments for the application of AIDER are daily life and clinic rehabilitation. Compared with the clinical environment, the daily life environment is more complex. Therefore, in this research, we pay more attention in analyzing the main features of the daily life environment, especially the ground. Generally, two main features of the ground influence the gait for normal walking, that is, hardness and terrain.  Figure 2 shows the relationship between these two features and the common implementation environment. The typical materials in daily life have two relative features: for example, the typical features of marble ground are flat and hard.

### 2.2. Gait Analysis and Environment Detection for the AIDER

For healthy people, the gait is changed adaptively when they cross from one terrain to another. For instance, the gait will change when someone crosses from hard ground to sand. However, the AIDER works on a customized gait to realize the walking motion for SCI patients [15]. It would cause a potential safety hazard because it cannot adapt to the change of terrains adaptively in a social environment.

Gait analysis mainly focuses on two parameters which are ground reaction force (GRF) and body posture. These two parameters can be utilized to confirm whether the system state is suitable for the next motion. For the stability control of biped robots, findings from [27] indicated that, comparing with a hard ground, step height tends to increase for avoiding collision between a robot's feet and soft ground. Besides, terrain features are also the essential factors for gait adjustment. For AIDER, the control strategy of stability not only depends on the system controller but also the environmental features. Typical environments for AIDER are shown in Figure 2. Therefore, we intend to design a wearable sensing system for AIDER which can be used to detect and recognize environmental features and CoP of the feet in this paper.

## 3. Method and Materials

### 3.1. Design Requirement of a Wearable Sensing System

Section 1 shows the benefit of a wearable sensing system for gait analysis and the safety of a human-machine system. Therefore, a wearable sensing system for the feet is proposed to realize gait analysis and environment detection. The following design requirements are proposed according to the features of a SCI patient:

- To make the user comfortable, the thickness of sole should be less than 20 mm.
- To ensure convenience, people should be able to put on the shoe using one hand.
- To ensure the accuracy for gait analysis, the magnitude of output force from the sensors should be obtained.
- To avoid the force from exceeding the acceptable range, the force measurement range of the wearable sensing system should be from 0 to at least 120 kg.
- To cut the cost, the hardware cost of the wearable sensing system should be less than ¥2000.
- To provide enough data for control strategies, the wearable sensing system should be designed to be able to detect and recognize ground features.
- Finally, the wearable sensing system needs to realize attitude measurement and gait analysis.

### 3.2. Design of the Wearable Sensing System

**Figure 3:**
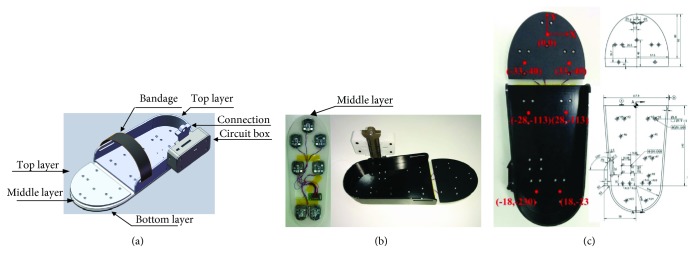
The mechanical structure of the wearable sensing system for AIDER. (a) The structure of the wearable sensing system. (b) The prototype of the wearable sensing system. (c) The coordinate system for the left foot.

As proposed in Section 3.1, 7 design requirements should be met. The mechanical design of the wearable sensing system is shown in Figure 3. In Figure 3(a), there are 3 layers that form the sensing part for force detection. The bottom layer is constructed of wear-resistant rubber which is used to ensure that the pilot's foot does not slip. A hook and loop tape is used to fasten the foot. The middle layer is a holder for the force sensor. The total thickness of the 3 layers is 18 mm which can meet the conditions of requirement (a). Seven strain gauge force sensors are employed to sense the center of force in the *z*-axis. The top layer is used to install the 7 force sensors which are made of aluminum alloy. It is necessary to recognize the force for the heel and forefoot [27, 28]. Therefore, the top layer is made of two separated aluminum alloys. Three force sensors on the forefoot form a stable plane. The other 4 force sensors form a trapezoid to keep stable. The accuracy of each force sensor is about 0.1%. All the seven force sensors together are capable of high accuracy measurement. Because the range of a force sensor is about 25 kgf, the measured range of the wearable sensing system is about 175 kgf. The IMU sensors and control circuit are used to collect attitude data which is installed in the circuit box as shown in Figure 3(a). The connection rod is designed to link the wearable sensing system and the shanks of AIDER. To meet requirement (e), the cost of the wearable sensing system is listed in Table 2.

### 3.3. Force Measurement Experiment

**Figure 4:**
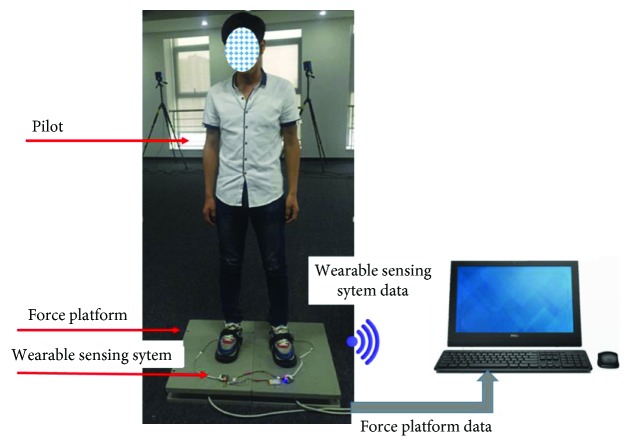
The setup of the stability test for a force measurement experiment.

**Figure 5:**
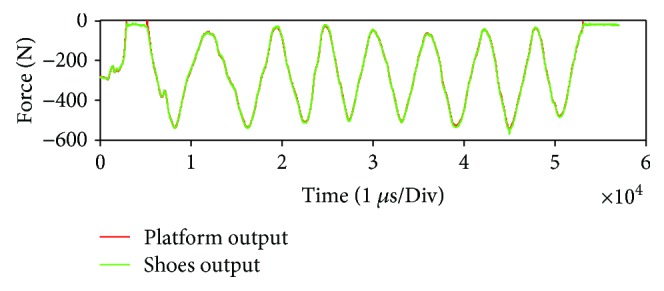
Results for the accuracy verification of a wearable sensing system.

To test the performance of our force sensing system in terms of accuracy and dynamic stability, a force measurement experiment was conducted. In this experiment, we used the force platforms to verify and calibrate the accuracy of the wearable sensing system.  Figure 4 describes the setup of the force measurement experiment, where the pilot stands astraddle on two force platforms. During the experiment, the pilot shifts the support foot at the center of his body weight from the left to the right and then shifts back again to the left. The motion frequency is about 2 seconds. Finally, we used a wireless module to translate the data of the wearable sensing system to a PC for sensing system analysis. The experimental results are indicated in Figure 5. The red line indicates the output of the force plates and the blue line indicates the output of the wearable sensing system. This figure shows that the data from the shoes follow the data from the force plate with high precision. With a shaking motion, the wearable system detected the body shaking accurately.

### 3.4. Center of Pressure (CoP)

For biped locomotion control, ZMP (zero moment point) and CoP are two important criteria. CoP coincides with ZMP when the system is under a quasistatic state. Di et al. developed a cane robot to realize human fall detection by estimating the CoP [29]. In our research, CoP is also involved in AIDER for stability estimation of the human-machine system. To estimate the CoP of the human-machine system, the first step is to calculate the ground reaction force (GRF) and the CoP of the feet. According to [30], the CoP can be estimated by

(1) $$\begin{array}{c} X_{CoP}=\frac{\int x\cdot F\left(x\right)dx}{\int F\left(x\right)dx},\\ Y_{CoP}=\frac{\int y\cdot F\left(y\right)dy}{\int F\left(y\right)dy}. \end{array}$$

Based on the mechanical design of the force sensory system, after being dispersed (1), the CoP of the foot is obtained by

(2) $$\begin{array}{c} X_{CoP}=\frac{\sum x_{i}\cdot f_{ni}}{\sum f_{ni}},\\ Y_{CoP}=\frac{\sum y_{i}\cdot f_{ni}}{\sum f_{ni}}, \end{array}$$

where *P*_*i*_(*x*_*i*_, *y*_*i*_), (*i* = 1, 2,…, 7) denotes the coordinate for each force sensor. *f*_*ni*_ denotes the force that is obtained by each force sensor. *n* is the mark for recognizing the left and right foot. Based on mechanical design, the coordinate of each sensor can be obtained by Figure 3(c).

**Figure 6:**
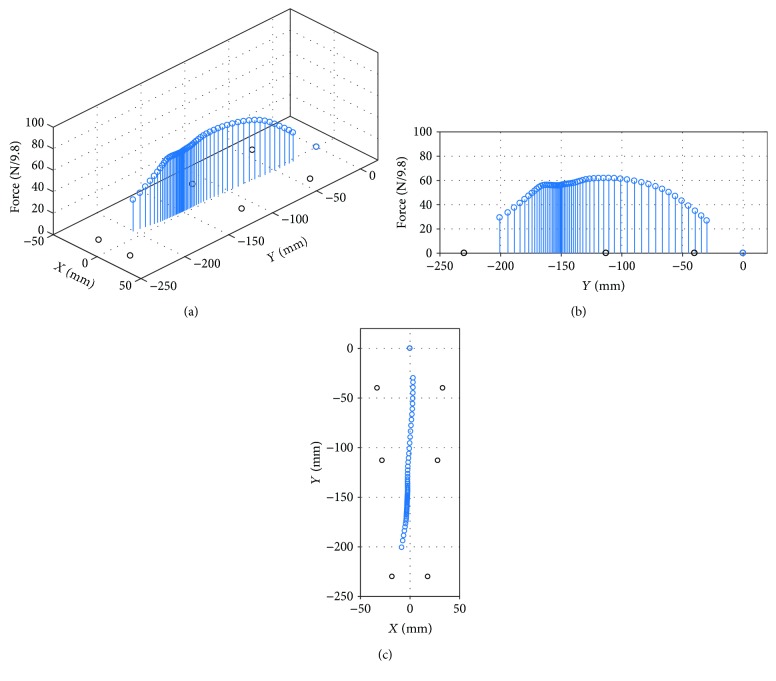
The CoP for the left foot; the black circle denotes the pressure-bearing point. (a) The trajectory of the CoP. (b) The magnitude of the total force during contact of the left foot to the ground. (c) The trajectory of CoP when the heel touches the ground up to point when the toe lifts from the ground.

A verification experiment is designed to prove the performance of a wearable sensing system for CoP detection. The experimental setup is similar to that in Figure 4. The difference is that the pilot is walking in a daily life state but on a force platform.  Figure 6 showed the experimental results from the point when the heel touches the ground up to the point when the toe lifts from the ground. In this experiment, the trajectory transforms from the heel to the big toe as shown in Figure 6(a).  Figure 6(b) shows the magnitude of the total force in the *z*-axis. The trajectory of CoP in the *X*-*Y* plane is shown in Figure 6(c). Based on [31], the trajectory of CoP agrees with human habit because of a similar curve.

## 4. Ground Characteristic Analysis and Recognition

### 4.1. Ground Characteristic Analysis

Based on Section 2, the main features of the application environment contain hardness and terrain. More specifically, soft/hard and flat/slope are two pairs of critical factors for a control strategy. After considering the environment in daily life, carpet, ramp, and marble ground are selected as the recognized subjects. For the ramp, uphill and downhill is the difference. For a flat ground, soft and hard is the main difference feature. Therefore, the main purpose of the wearable sensing system is to recognize the following combined features which are flat/hard (F/H), flat/soft (F/S), uphill/hard (U/H), and downhill/hard (D/H).

To recognize these features, the data from the IMU and force sensor are necessary. Generally, a walking motion can be divided into 8 phases, that is, initial contact (IC), loading response (LR), midstance (MS), preswing (PS), initial swing (IS), midswing (MS), and terminal swing (TS) [24]. For a ramp, 3 force sensors are used in [32] to recognize the slope by adjusting the sequence of the force sensor output. The IC and LR phases contain impact information caused by the hardness of the ground. Therefore, the sensor data from the IC and LR phases are collected by the data window for feature recognition.

**Figure 7:**
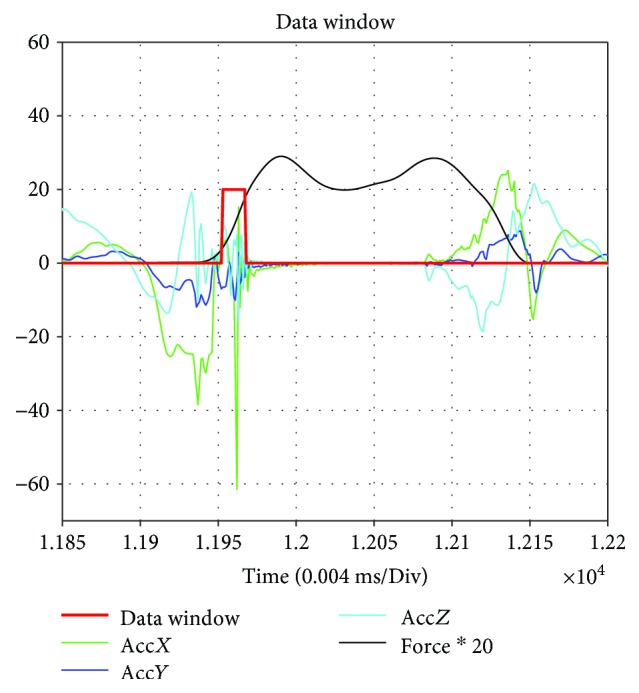
Data window for the feature extraction. The force multiplied by 20 is the aim for analyzing in the same magnitude.

A total of 7 force sensors and one IMU is used to sense the ground features. The force of each sensor is *f*_*ni*_ (*i* = 1, 2,…, 7). The output of the IMU sensor is angular velocity $\omega ={\left[\begin{array}{ccc} \omega_{x} & \omega_{y} & \omega_{z} \end{array}\right]}^{T}$ and acceleration $a={\left[\begin{array}{ccc} a_{x} & a_{y} & a_{z} \end{array}\right]}^{T}$. To get a credible result, the gravitational acceleration is removed and the resultant force of the 7 sensors is normalized. To keep the data in the same magnitude, the force is multiplied with a scale factor. The drastic vibration makes the IC and LR phases easy to detect and the force output also increases. Therefore, according to the output of the force sensors, the data window for feature recognition is obtained as shown in Figure 7.

### 4.2. Principal Component Analysis (PCA) for the Four Ground Feature Extraction

PCA is a data analysis method that uses an orthogonal transformation to obtain principal components which are used to present the original data feature by a low-dimensional variable. As a popular pattern recognition method, PCA is widely used in face recognition [33]. This method is aimed at reducing the dimension for the eigenvector. In our research, the dimension of the eigenvector for an environmental feature extraction is 30 which contains the sum, mean, and variance of the 7 normalized force sensor output and 3-axis motion acceleration. After analyzing the data by PCA, the variance that explains principal components are obtained.

**Figure 8:**
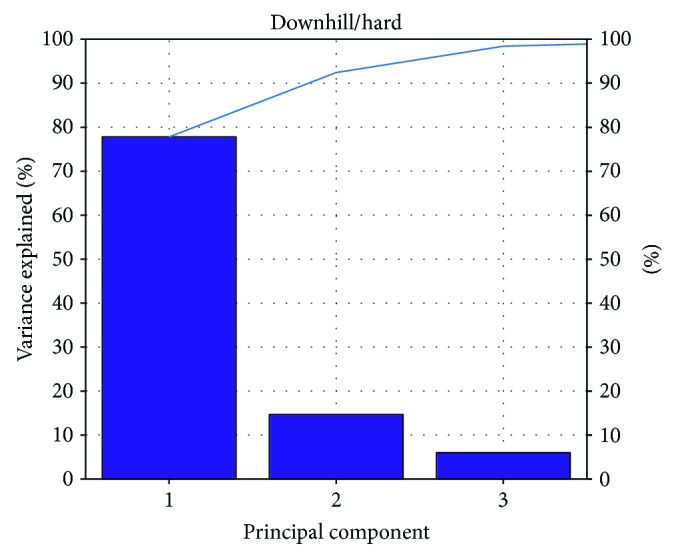
The relationship between the principal component and the variance explained for D/H.

**Figure 9:**
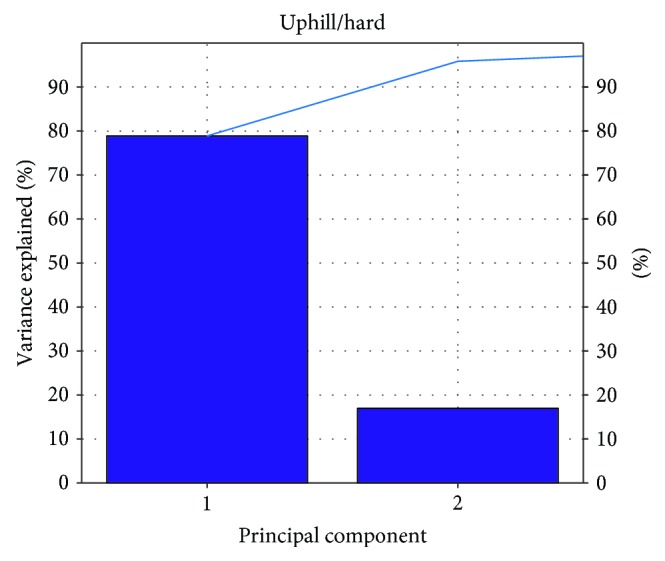
The relationship between the principal component and the variance explained for U/H.

**Figure 10:**
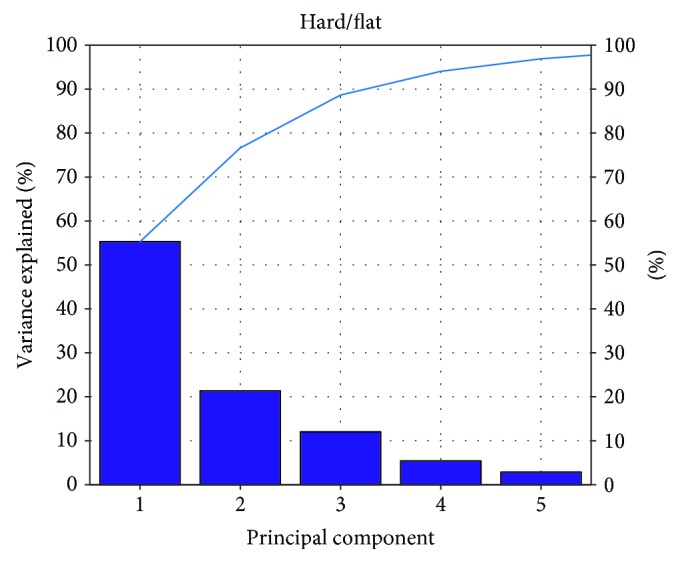
The relationship between the principal component and the variance explained for H/F.

**Figure 11:**
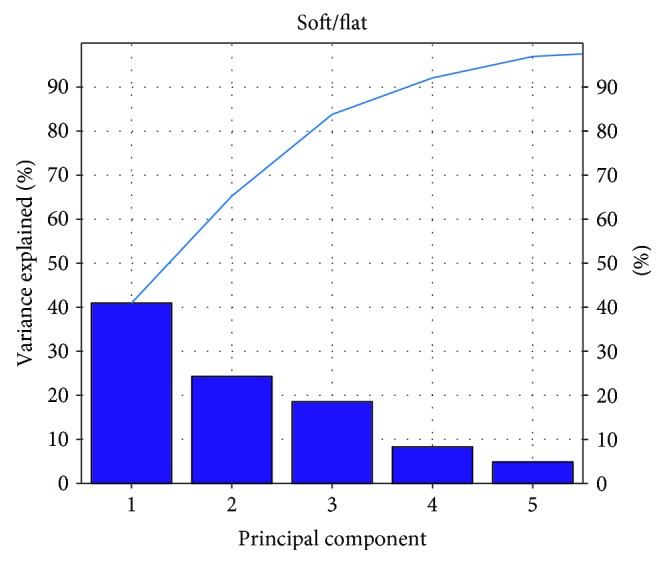
The relationship between the principal component and the variance explained for S/F.

**Figure 12:**
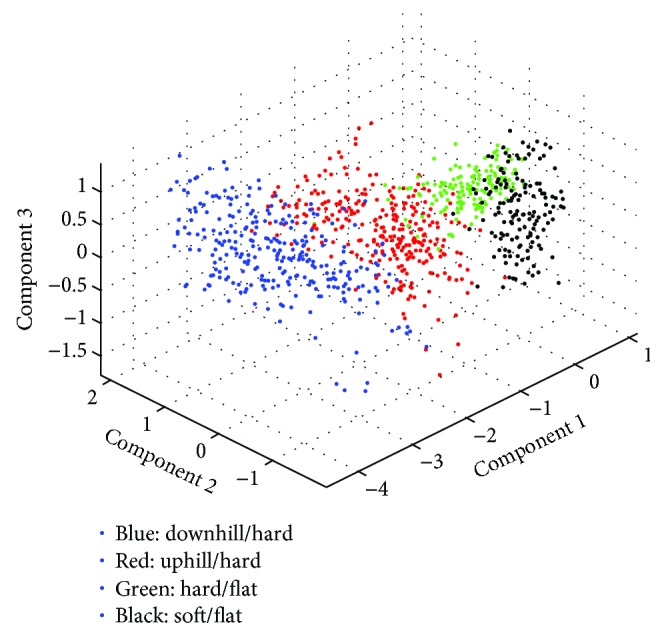
The four ground features are described by the first three principal components.

**Figure 13:**
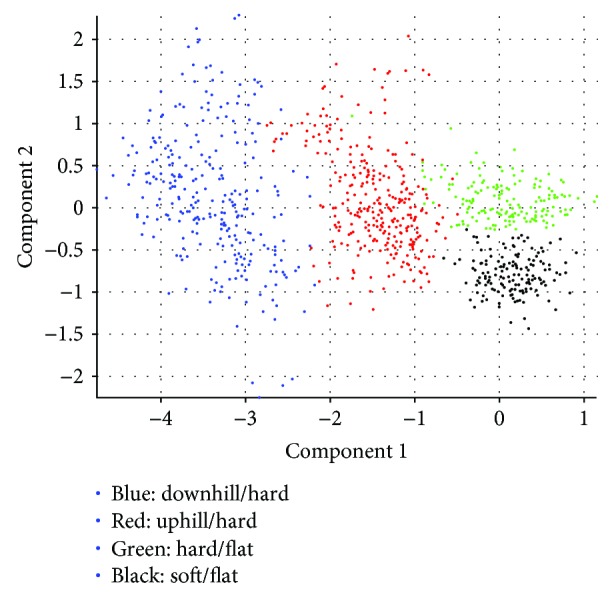
The four ground features are described by the first two principal components.

According to the result of Figures 8–11, the variance that explained the first three principal components is more than 85%. Therefore, the first three principal components are enough to distinguish the four ground features. Figures 8 and 9 particularly show that the uphill and downhill motions are easy to describe using the first 2 principal components. In Figures 9 and 10, the variance explained on the third principal component is more than 10%. The results indicate that the soft and hard features are relatively complex. Finally, the ground features are described by the first three and two principal components in Figures 12 and 13, respectively.

Due to the four ground features, it is easy to classify the minimum-distance classifier [34] which is employed to classify the four features.

### 4.3. Experiments

**Figure 14:**
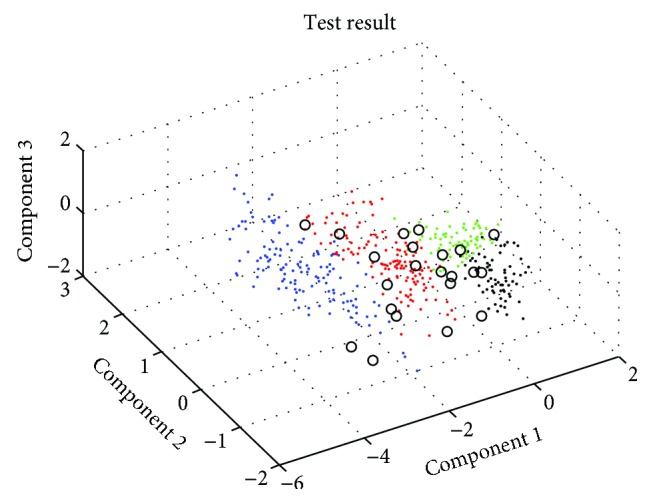
The recognition results for the 4 ground features.

To verify the performance of the recognition method, 5 experimental subjects wore the wearable sensing system and walked on carpet, ramp (uphill and downhill), and flat ground surface in a normal gait, and about 2000 steps were obtained. After preprocessing, half of data are used as training data. The left part is used for testing the training model, and the experimental results are obtained as shown in Figure 14. The black circles indicate the points that are not classified in features on the right.

Finally, the recognition rate for 4 ground features is listed in Table 3. The recognition rate of hard/flat ground and downhill/hard ground is more than 95%. This result shows that the eigenvector which is extracted by PCA and the minimum-distance classifier is suitable for the ground feature recognition.

## 5. Discussion

A mechanical design has been proposed in Section 3.2 that meets design requirements (a), (b), (d), and (e). To meet design requirement (c), 7 force sensors were used to form a force measurement plate. The force sensor can bear the weight of a pilot. The main contribution of this research is that ground feature detection and recognition were realized which is mentioned in requirements (f) and (g). Attitude and force data were combined to get the data window which is used to analyze the ground features. Besides, the main work of the IMU is to obtain the attitude data for the shoes. The detection result is also the effect of the properties of the material used in the bottom layer. The rubber layer can absorb the noise from the motion of touching the ground.

PCA and the minimum-distance classifier are involved in realizing the ground feature recognition. Four classical terrains are involved in this paper. However, the daily life environment is more complex than an experiment. The data in Table 3 indicates the accuracy of the ground feature recognition. The maximum error is about 5.3% which occurred on soft and hard ground feature detection. An error of about 4.4% occurred in the up and down features.

## 6. Conclusions and Future Work

In this work, we introduced the application environment for AULLE, RELLE, and ASLLE respectively. As an ASLEE, AIDER is used to help SCI patients return to a normal life. We proposed a wearable sensing system that is able to improve the flexibility and safety of the pilot by detecting gait and GRF. The mechanical design of the wearable sensing shoes is proposed to fulfill the design requirements. Seven force sensors are used to form two rigid planes to detect the GRF that showed a good performance on force and CoP detection. The IMU sensor was installed on the wearable sensing system to sense the attitude and acceleration data which were calculated for ground feature recognition. A verification experiment of the wearable sensing system was executed to test the performance for CoP detection. The results indicated that the wearable sensing system is able to realize human gait trajectory detection, and the trajectory trend of CoP agrees with the normal human trajectory. PCA is involved in ground feature recognition because of the large dimension of the eigenvector. The analysis result showed that the first three principal components are enough for the uphill/hard, downhill/hard, hard/flat, and soft/flat ground feature extraction. Finally, a test was carried out to verify the recognition performance, and the results showed that the recognition rate is more than 93%.

In the future, more environmental situations should be considered into the recognition experiment to verify the performance of the wearable sensing system, for example, a road made of sand and cobblestones. Until now, the recognition algorithm is still executed on a PC, which is not convenient for real time work.

## Figures and Tables

**Table 1:** The influence for three kinds of exoskeletons on the human-machine-environment.

Factors	AULLE	RELLE	ASLLE
Human	Health	Weak	Weak
Machine	Low	High	High
Environment	Daily life/unsafe	Hospital/safe	Daily life/unsafe

**Table 2:** The cost of wearable sensing system.

Name	Unit cost	Unit	quantity	Total price/¥
IMU	25	piece	2	50
Force sensor	15	piece	14	210
Mechanical parts	500	Set	2	1000
Circuit board	100	Piece	4	400
Hook and loop tape	10	piece	2	20
Sum				1680

**Table 3:** The recognition rate for the 4 features.

	D/H	U/H	H/F	S/F
D/H	95.620%	4.380%	0	0
U/H	0.680%	93.878%	2.721%	2.721%
H/F	0	3.448%	96.552%	0
S/F	0	0	5.333%	94.667%

## Data Availability

The data used to support the findings of this study are available from the corresponding author upon request.
